# Fully Automated Enhanced Tumor Compartmentalization: Man vs. Machine Reloaded

**DOI:** 10.1371/journal.pone.0165302

**Published:** 2016-11-02

**Authors:** Nicole Porz, Simon Habegger, Raphael Meier, Rajeev Verma, Astrid Jilch, Jens Fichtner, Urspeter Knecht, Christian Radina, Philippe Schucht, Jürgen Beck, Andreas Raabe, Johannes Slotboom, Mauricio Reyes, Roland Wiest

**Affiliations:** 1 Support Center for Advanced Neuroimaging—Institute for Diagnostic and Interventional Neuroradiology, University Hospital Inselspital and University of Bern, Bern, Switzerland; 2 Department of Neurosurgery, University Hospital Inselspital and University of Bern, Bern, Switzerland; 3 Institute of Surgical Technology and Biomechanics, University of Bern, Bern, Switzerland; University of Pennsylvania, UNITED STATES

## Abstract

**Objective:**

Comparison of a fully-automated segmentation method that uses compartmental volume information to a semi-automatic user-guided and FDA-approved segmentation technique.

**Methods:**

Nineteen patients with a recently diagnosed and histologically confirmed glioblastoma (GBM) were included and MR images were acquired with a 1.5 T MR scanner. Manual segmentation for volumetric analyses was performed using the open source software 3D Slicer version 4.2.2.3 (www.slicer.org). Semi-automatic segmentation was done by four independent neurosurgeons and neuroradiologists using the computer-assisted segmentation tool SmartBrush® (referred to as SB), a semi-automatic user-guided and FDA-approved tumor-outlining program that uses contour expansion. Fully automatic segmentations were performed with the Brain Tumor Image Analysis (BraTumIA, referred to as BT) software. We compared manual (ground truth, referred to as GT), computer-assisted (SB) and fully-automated (BT) segmentations with regard to: (1) products of two maximum diameters for 2D measurements, (2) the Dice coefficient, (3) the positive predictive value, (4) the sensitivity and (5) the volume error.

**Results:**

Segmentations by the four expert raters resulted in a mean Dice coefficient between 0.72 and 0.77 using SB. BT achieved a mean Dice coefficient of 0.68. Significant differences were found for intermodal (BT vs. SB) and for intramodal (four SB expert raters) performances. The BT and SB segmentations of the contrast-enhancing volumes achieved a high correlation with the GT. Pearson correlation was 0.8 for BT; however, there were a few discrepancies between raters (BT and SB 1 only). Additional non-enhancing tumor tissue extending the SB volumes was found with BT in 16/19 cases. The clinically motivated sum of products of diameters measure (SPD) revealed neither significant intermodal nor intramodal variations. The analysis time for the four expert raters was faster (1 minute and 47 seconds to 3 minutes and 39 seconds) than with BT (5 minutes).

**Conclusion:**

BT and SB provide comparable segmentation results in a clinical setting. SB provided similar SPD measures to BT and GT, but differed in the volume analysis in one of the four clinical raters. A major strength of BT may its independence from human interactions, it can thus be employed to handle large datasets and to associate tumor volumes with clinical and/or molecular datasets ("-omics") as well as for clinical analyses of brain tumor compartment volumes as baseline outcome parameters. Due to its multi-compartment segmentation it may provide information about GBM subcompartment compositions that may be subjected to clinical studies to investigate the delineation of the target volumes for adjuvant therapies in the future.

## Introduction

Volumetry of malignant brain tumors (glioblastoma multiforme (GBM)) is usually performed semi-automatically or by manual delineation by an expert in a clinical setting. The latter option is hampered by time and personnel costs, as well as by inter-rater variability [1]. Volumetry is frequently integrated into treatment planning (e.g. to inform the neurosurgeon about tumor location for operation planning, and radiation oncologists to support therapy planning [2–4]). GBM subcompartment volume analysis may aid in biophysical modeling of brain tumor infiltration [5]. Appropriate assessment of the extent of resection plays a role in the prognosis of GBMs, since maximizing the extent of resection influences survival in glioblastoma patients. A complete resection of enhancing tumor, defined as the removal of the final 1–2% of the tumor, seems to provide the most benefit in terms of survival [6–8]. Further, there is increasing evidence that tumor expansion beyond areas of blood–brain barrier disruption (i.e. the enhancing compartment) impacts survival of patients with a GBM [9–11] and should be considered in pre-surgical planning [12].

Fully-automated user-independent segmentation tools are currently employed predominantly for research [13]. Semi-manual image-guided contouring software is routinely used in many operating theaters and in radiation oncology, but still requires user-dependent manual interaction.

We recently devised the fully-automated multimodal segmentation tool BratTumIA (BT) for pre-surgical and longitudinal tumor segmentation [14, 15]. BT calculates tumor volumes of healthy and tumor tissue compartmentalization with a variability that is comparable to that achieved with time-consuming manual tumor delineation by an expert [13, 14]. In this study, we aimed to investigate the performance of fully- vs. semi-automatic brain tumor volumetry. For comparison, we employed SmartBrush® (SB), which is routinely used for surgical planning and volumetric quantification of pre- and postoperative tumor volumes [9, 16]. We determined whether: (i) 2D diameter-based and (ii) 3D volumetric-based criteria for brain tumor assessment can be reliably calculated with BT and SB, (iii) whether the (semi-)automatic segmentations are comparable with an expert-rater-based ground truth (GT) in terms of the Dice coefficient, and (iv) whether the subcompartment analysis by BT, which takes into account the infiltrative growth patterns of GBMs, improves the routine estimations of gross tumor volume.

## Materials and Methods

### Study population

Patients with a newly diagnosed and histologically confirmed GBM, enrolled preoperatively at our institution between October 2012 and July 2013, underwent prospective manual segmentation, semi-automatic and automated volumetry. The manual GT was previously used in part by Porz et al. [15].

Exclusion criteria were: incomplete image acquisition, Karnofski performance status < 70%, abnormal hematologic, renal or hepatic function, and previous cranial neurosurgery. The study was approved by the Local Research Ethics Commission “Kantonale Ethikkommision Bern”. All patients provided written informed consent.

### MR imaging protocol

MR images were acquired with similar protocols and field strengths on two 1.5 T MR scanners from one vendor (Siemens Avanto and Siemens Aera, Siemens, Erlangen, Germany). Every patient was subject to a standardized MR imaging protocol including: (1) pre-contrast 3D-T1w-multiplanar reconstruction (MPR) in sagittal acquisition, 1 mm isotropic resolution; (2) post-contrast 3D-T1w-MPR in sagittal acquisition, 1 mm isotropic resolution; (3) 3D-T2w (SPC) in sagittal acquisition, 1 mm isotropic resolution; and (4) fluid-attenuated inversion recovery (FLAIR) (2D turbo inversion recovery) in axial acquisition. The sequence parameters were: (1) for pre-contrast 3D-T1w MPR sequences echo time (TE) = 2.67 ms, repetition time (TR) = 1580 ms, field of view (FOV) = 256 × 256 mm^2^, flip angle (FA) = 8°, with an isotropic voxel resolution of 1 × 1 × 1 mm; (2) for post-contrast T1w TE = 4.57 ms, TR = 2070 ms, FOV = 256 × 256 mm^2^, FA = 15°, using isotropic 1 × 1 × 1 mm voxels; (3) for 3D-T2w (SPC) in sagittal acquisition TE = 380 ms, TR = 3000 ms, FOV = 256 × 256 mm^2^, FA = 120°, using isotropic 1 × 1 × 1mm voxels; (4) for 2D FLAIR sequence TE = 80 ms, TR = 8000 ms, FOV = 256 × 256 mm^2^, FA = 120°, using a non-isotropic voxel size of 1 × 1 × 3 mm.

### Comparative metrics

Different metrics were employed to assess the segmentation quality of the two methods and of the four SB raters. The quantitative measures computed included Dice coefficient [17], absolute and relative volume, sum of products of squared diameters (SPD), sensitivity and positive predictive value (PPV). The Dice coefficient is a value between zero and one that expresses the amount of overlap between two segmentations, with one being a perfect overlap. It can be considered a standard metric in image analysis [17]. Besides the volumes of the individual segmentations, the relative volume, which is the segmented volume subtracted from the GT volume, was also evaluated. The SPD metric was mainly considered due to its prevalence in clinical practice, which in turn is due to its capability to provide a fast assessment of the tumor growth rate. Moreover, the World Health Organization recommends use of the SPD for gross tumor volume [18] and refined response assessment [19]. The segmentation sensitivity states what proportion of actual tumor was detected by the method and/or rater [20]. Last but not least, the PPV denotes the correctly segmented tumor divided by segmented tumor [21]. Sensitivity and PPV again yield values in the range between zero and one. Values closer to one indicate a better performance.

Although the objective of this study was to compare a fully-automatic brain tumor segmentation method with a commercially available semi-automatic one, it was decided not to merge the data from the four SB raters. Segmentation variability is always an issue one has to consider when dealing with methods that require human interaction and should therefore not be neglected in a comparative study. Furthermore, the decision was taken on the basis that no merging method should be applied unless out of necessity with respect to the main objective of the study, as such merging is always subject to data loss. Finally, the decision is supported by the results obtained during this study and by [22] where issues with the widely used simultaneous truth and performance level estimation (STAPLE) merging algorithm are discussed.

### Manual volumetry

**Fig 1:**
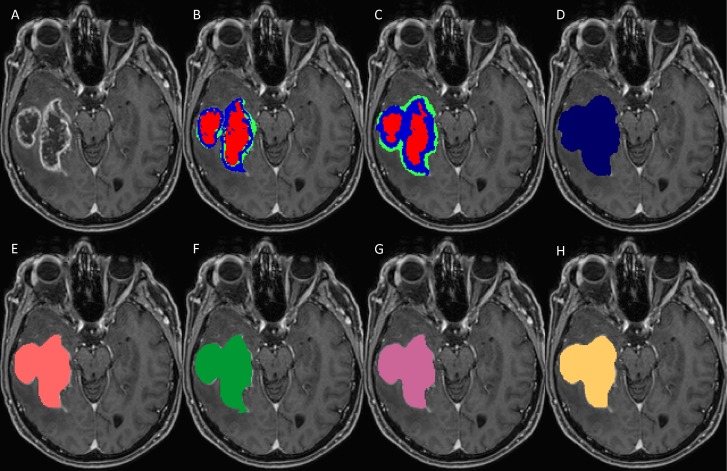
Set of MRI sequences used in this study for manual, automatic, and semi-automatic tumor volumetry. Original T1-weighted post-contrast MRI slice (A), manual subcompartmental segmentation into non-enhancing tumor (green), enhancing tumor (blue), and necrotic tissue (red) (B). BT subcompartmental segmentation into non-enhancing tumor (green), enhancing tumor (blue) and necrotic tissue (red) (C). BT core tumor segmentation (dark blue, D), SB1 core tumor segmentation (light red, E), SB2 core tumor segmentation (green, F), SB3 core tumor segmentation (purple, G) and SB4 core tumor segmentation (yellow, H).

Manual segmentation was performed by a neurosurgeon experienced in brain tumor analysis and supervised by a neuroradiologist with more than 15 years of experience in brain tumor imaging. The neurosurgeon employed the open source software 3D Slicer Version 4.2.2.3 (www.slicer.org) [23]. The images from the 19 patients were segmented manually slice by slice (Fig 1(B)). Segmentation was performed on T1w, T1wGd (Fig 1(A)), T2w and FLAIR sequences according to the VASARI MR feature guide v.1.1 (https://wiki.nci.nih.gov/display/CIP/VASARI). For intermodal comparisons and analysis, the gross tumor volume (TV)–encompassing the enhancing part, the non-enhancing part and the necrotic core of the GBM–was selected. Manual segmentation was defined as the GT for further analyses [13]. Note that in the rest of this article we use GT and manual segmentations interchangeably.

### Automated segmentation

The fully automatic segmentations of this study were performed using BT (https://www.nitrc.org/projects/bratumia/). BT integrates a pipeline of three distinct parts, namely the preprocessing, classification and regularization units. The software takes as its input the widely acquired structural T1, T1-contrast (T1w), T2 and FLAIR MRI sequences. The preprocessing unit aligns these sequences to a common position and resolution through registration [24]. Also, a brain extraction mask computed from the T1w volume is applied to the four sequences. The output is forwarded to the classification unit. For every voxel in each volume a number of features, such as the mean intensity in its neighborhood, are computed. A random forest classifier then decides, based on the features exhibited by a voxel, which tissue type it depicts. The last unit is necessary to reduce implausible or even impossible tissue constellations between neighboring voxels. The core of BT evolved from [25] and the methodology was previously described in [14]. BT is able to distinguish seven brain tissue types, three healthy (gray matter, white matter and cerebrospinal fluid) and four tumor (edema, necrosis, non-enhancing tumor and enhancing tumor) tissues (Fig 1C and 1D; healthy tissues are not shown). Along the segmentations, BT further outputs the skull-stripped structural sequences and a report file with, among other information, the individual tissue volumes and the SPD value.

**Fig 2:**
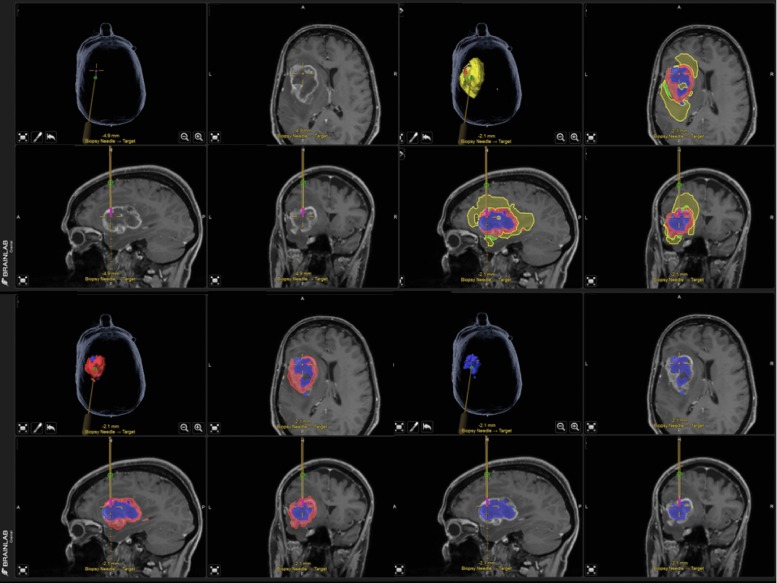
Stereotactic biopsy with the frameless neuronavigation system (Brainlab® VarioGuide) using BraTumIA segmentation. The figures indicate the T1w raw image and the BT subcompartment overlays during biopsy. Upper row left column: original T1wGd without tumor delineation, right column: all automatic segmented tumor subcompartments are visible. Bottom row left column: necrosis and contrast-enhancing tumor volume, right column: only necrotic tumor volume. Color code for segmentations: red = enhancing tumor, yellow = edema, blue = necrosis and green = non-enhancing tumor.

To illustrate the functionality and potential application of BT, we present the example of its additional use during biopsy in a GBM case. Biopsy was performed with the frameless neuronavigation system (Brainlab® VarioGuide). BT segmentations were loaded to the iPlan (3.0.2 cranial) software as an overlay to the post-contrast T1 MRI sequence. The results of the subcompartment analysis (i.e. of the enhancing part, the non-enhancing T2/FLAIR-hypointense part, the T2/FLAIR hyperintense vasogenic edema and the necrotic core) were stored in Brainlab® native object format. During surgery, it is possible to add the subcompartment segmentation objects as an overlay to the original MRI sequence, according to the surgeon’s requirements (see Fig 2).

### Manual segmentation using SB

The semi-automatic segmentation was performed by four expert raters, three neurosurgeons (SB1, SB2 and SB4) and one neuroradiologist (SB3) (Fig 1E and 1H). All the neurosurgeons had more than five years of experience in semi-automatic brain tumor analysis and the neuroradiologist had more than five years of experience in MR reading of gliomas. All manual segmenters were instructed by an expert neuroradiologist with more than 15 years of experience in brain tumor imaging. The raters employed the contour-expansion-based, semi-automatic SB. It utilizes an intelligent region growing algorithm which, guided by the user, extends the brushed area to neighboring areas with similar intensities. The tumor has to be segmented in this manner on at least two slices in two different views. This provides sufficient input for the tool to compute the corresponding 3D tumor volume. The result can be further improved by extending or erasing the computed segmentation on individual slices.

### Statistical methods

For a first overview with a one-way analysis of variance, the non-parametric Kruskal-Wallis test was applied due to the non-normally distributed character of the data. The pairwise significance test was performed using the two-sided Wilcoxon signed-rank test [26]. The p-values were corrected for multiple comparisons with the Bonferroni method [27]. The chosen significance level was α = 0.05%. The interrelationship between the manual, semi- and fully-automatic segmentations was determined with the Pearson correlation coefficient. The statistical analysis was carried out with RStudio (http://www.rstudio.com).

## Results

### Study population

The mean age of patients at preoperative MR imaging was 65 years (range 37–76 years) and the mean pre-operative Karnofski performance status was 85 ± 26% (range 70–90%). Nine of the 19 patients were female. Five patients underwent stereotactic biopsy, six subtotal resections and eight complete resections of enhancing tumor. All diagnoses were confirmed by histopathology.

### SPD

**Fig 3:**
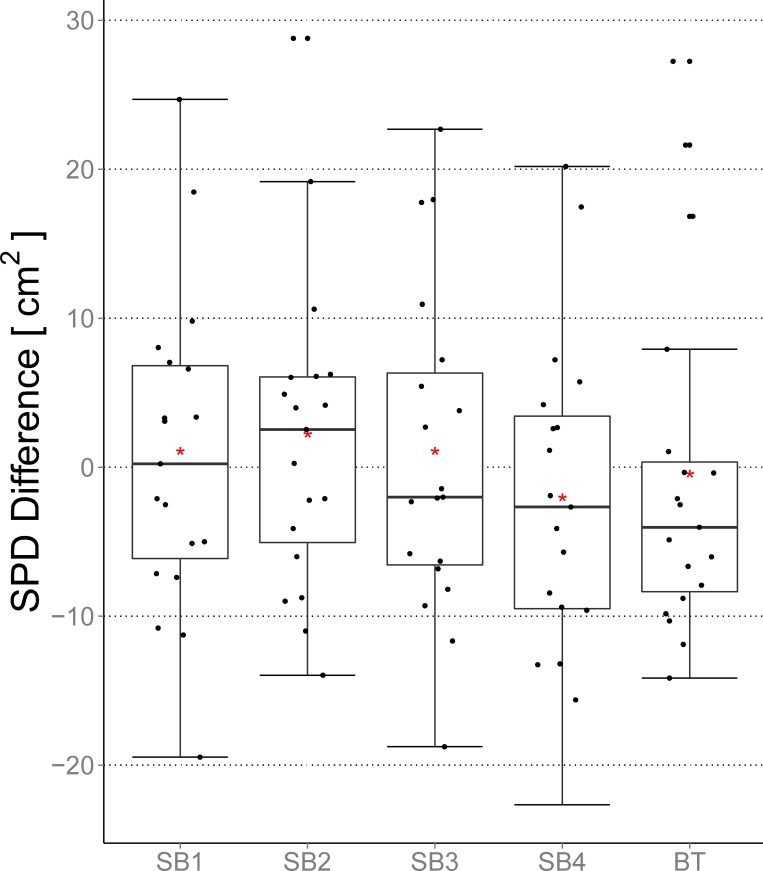
Differences between the SPD metric and the GT for the BT and the four SB segmentations. Additionally to the general boxplot statistics the mean value is shown (red asterisk). Negative values imply an overestimation by the rater/method whereas positive values indicate an underestimation.

The differences between the SB/BT segmentations and the GT are depicted in Fig 3. BT achieved a smaller interquartile range (IQR) than the four SB raters did. The median difference from the GT was lower for the SB segmentations. The rather large difference between median and mean (red asterisk) for SB3 and BT hints at a skewed distribution of the data (SPD differences) and/or strong outliers. In contrast, the differences in SPD for SB1, SB2 and SB4 appeared to be evenly distributed. No rater or method introduced strong outliers indicating overestimation, whereas SB2 and BT included underestimation outliers. Only SB2 showed a tendency to underestimate the SPD value. SB1 showed, in general, no such tendency. SB3 and SB4 as well as BT were more inclined to overestimate the 2D SPD measure.

**Fig 4:**
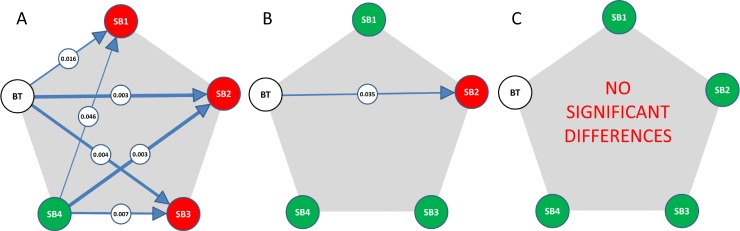
**The performances of the different raters (SB1 to 4) and methods (SB vs. BT) were compared with a Bonferroni corrected Wilcoxon signed-rank test in terms of Dice coefficient (A), absolute volume difference (B) and absolute SPD difference (C)**. A connection denotes a significant difference, with the encircled number being the p-value. The arrow points to the superior method with respect to the GT. The line thickness depicts the p-value in a qualitative manner. The color coding shows to which SB rater BT (white) is significantly different (red) or not (green).

Differences between the expert raters and methods were not significant (Fig 4).

### Dice coefficient

**Fig 5:**
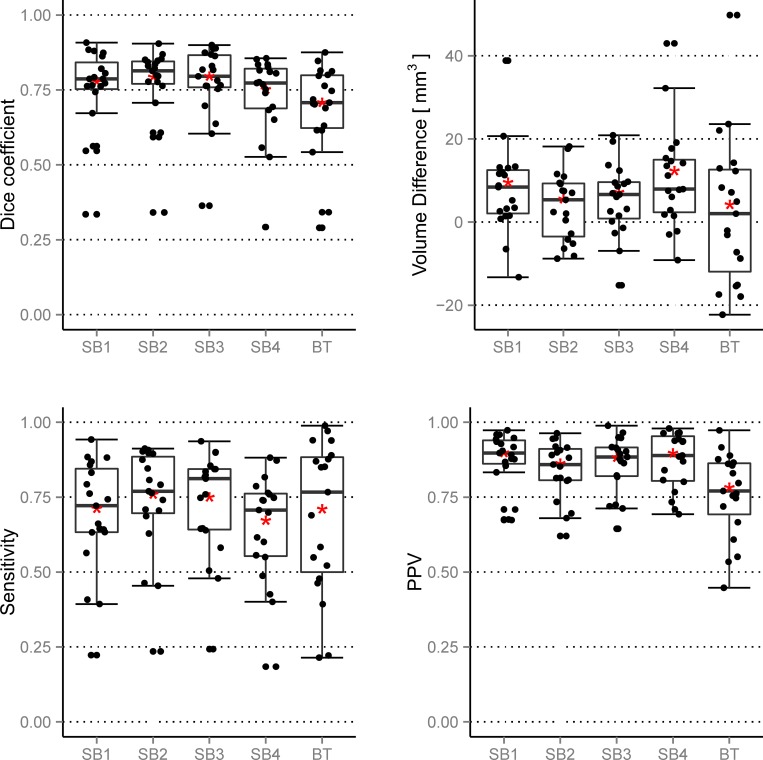
Left to right, top to bottom: Dice coefficient, volume difference, sensitivity and PPV. The results are obtained when BT and the four SB raters are compared to the GT. The figure depicts the general boxplot statistics with the additional mean value (red asterisk). For the volume differences, negative values denote overestimation and positive values underestimation compared to the GT.

The analysis of the Dice coefficient indicated a mean GT overlap of the four SB raters between 0.72 and 0.77 and of 0.68 for BT. An overview of these results is provided in Fig 5. The SB4 segmented similarly to BT. The outliers were similar for the SB1, SB2 and SB3, but differed from those of SB4 and BT.

The statistical difference or similarity of the raters/methods in terms of Dice coefficient is depicted in Fig 4(A). One can observe that there was a significant inter-rater variability during semi-automatic segmentation. The qualitative observation that rater SB4 was closest to BT was quantitatively confirmed with the result shown in Fig 4(A) (no connection between BT and SB4).

### Volume

**Fig 6:**
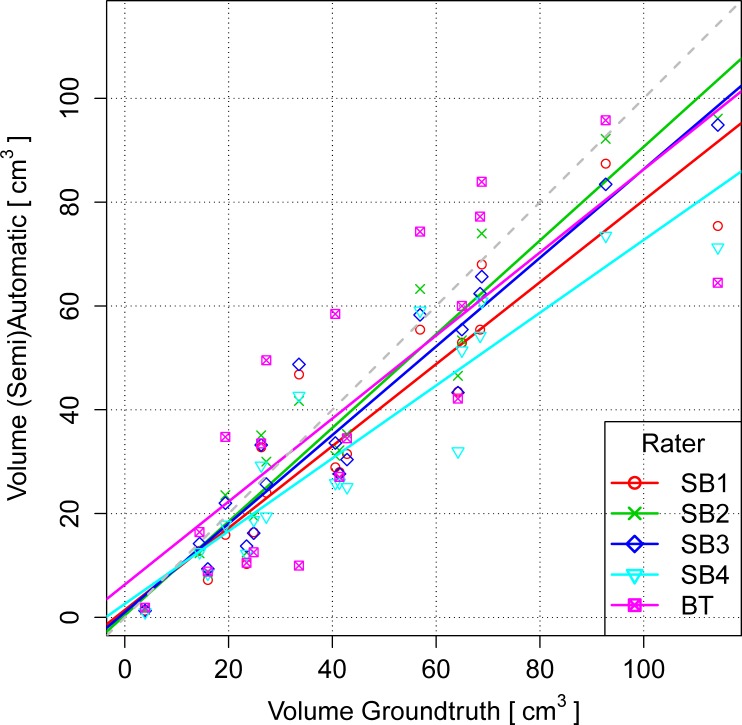
Volumes of the BT and the four SB segmentations (y-axis) plotted against the respective GT segmentations (x-axis). Perfect agreement with respect to tumor volume means that all data points (volumes) would come to lie on the gray dashed 45-degree line starting from the origin (0,0).

**Fig 7:**
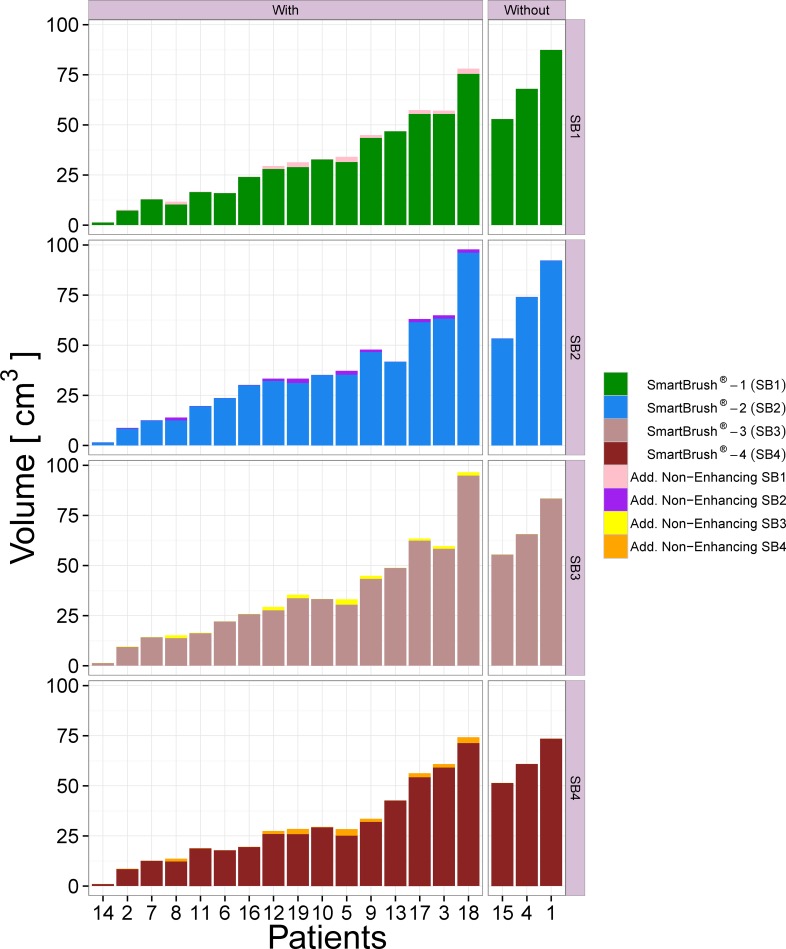
Barplot depicting the SB tumor volumes for the individual patients. Non-enhancing tumor tissue that was, as confirmed by the GT, correctly segmented with the multi-compartment BT software and was not part of the SB segmented volume, is stacked on top of them. The barplot is horizontally split into two groups of patients with and without additional non-enhancing tissue found by BT. Vertically, the figure is quartered to show the results for all four SB raters.

The volume differences to GT are illustrated in Fig 5. For BT we identified no systematic bias toward over- or underestimation of the volume (mean/median close to zero). SB raters tended to underestimate the volumes (see Fig 6). The volumes were tested for significant differences between all ten possible combinations of raters and methods. The result is depicted in Fig 4(B). A weakly significant difference (p-value rather close to α = 0.05%) could be found only for BT vs. SB2. As a next step, we analyzed the volumes of additional non-enhancing tumor tissue as provided by the subcompartment analysis of BT compared to the SB volumes (cf. Fig 7). In 16 out of 19 patients BT detected non-enhancing tumor. The correlation matrix of the segmented tumor volumes is provided in Table 1. All computed correlations were rather high, with 0.8 being the lowest–for BT vs. GT. The semi-automatic SB method achieved a high inter-rater and GT correlation, although the latter value was overall slightly inferior.

**Table 1:** Correlation Matrix for Automatically (BT), Semi-Automatically (SB) and Manually Derived Tumor Volumes.^a^

	SB1	SB2	SB3	SB4	BT	GT
SB1	1.00	0.99	0.98	0.99	0.85	0.93
SB2	0.99	1.00	0.99	0.98	0.86	0.96
SB3	0.98	0.99	1.00	0.98	0.83	0.96
SB4	0.99	0.98	0.98	1.00	0.85	0.92
BT	0.85	0.86	0.83	0.85	1.00	0.80
GT	0.93	0.96	0.96	0.92	0.80	1.00

^a^The Pearson correlation coefficient was employed.

### Sensitivity and PPV

BT achieved a high median sensitivity. The IQR was larger for BT than for SB and similar for all four SB raters (see Fig 5). The PPV of the SB raters showed good agreement, yet differed from BT. All raters and methods agreed on an even distribution of the PPV among patients. All four SB raters attained higher median PPV than sensitivity values, whereas BT appeared to be more consistent between PPV and sensitivity.

## Discussion

This study aimed to compare semi-automatic with fully-automatic brain tumor segmentation methods in terms of more clinically (SPD, volume) vs. technically relevant (Dice coefficient, PPV, sensitivity) metrics. While SB tended to be superior with respect to the metrics employed, we observed a discrepancy between technically and clinically applicable measures. The Dice coefficient, a standard metric in image analysis, reveals statistically significant differences not only between methods (BT vs. SB) but also between raters (four SB raters) of the same method. For the clinically relevant measures of the tumor volume, we observed only minor differences for one of the four raters, while the SPD calculations were in a comparable range.

SB can be reliably used for gross tumor segmentation as shown in [28]. However, inter-rater variability must be taken into account and is a principal drawback of user-dependent methods. Whether these variations primarily arise from selection bias of the perpendicular slices needed for interpolation, the variability of tumor delineation on these slices or the discrepancies in correction steps taken subsequently, is beyond the scope of this study. For BT, rater independence is an unarguable advantage in terms of segmentation consistency. This is of particular importance if tumor growth has to be addressed, e.g. in the framework of longitudinal clinical studies, pre-therapeutic tumor growth assessment before radiotherapy planning or for radiomics [29, 30]. Here, the subcompartment analysis of BT allows the integration of diffuse growth patterns beyond the disrupted blood–brain barrier that encompass the NETV. The automatic segmentation tool enabled visualization not only of the enhancing, but also of the T2/FLAIR-related GBM expansion within a single analysis. In this study, we observed extended NETV in 16/19 patients (confirmed by the GT), that were obscured by the SB segmentations. Intraoperative 5-aminolevulinic acid (5-ALA) fluorescence staining indicated that resection of non-enhancing tumor tissue may be useful in terms of overall survival, and that non-enhancing tumor compartments may be indicative of infiltrative and progressive tumor behavior [9, 11, 31, 32]. Non-enhancing tumor compartments were recently correlated with outcome and showed an impact of non-enhancing tumor on overall survival [16, 33]. As a consequence, the additional information provided by BT might further impact biopsy and resection planning, therapy selection [34–36] and monitoring intervals [37].

Tumors may consist of various heterogeneous tissue types that are shown by BT and that may improve knowledge of the heterogeneous cellular characteristics within the subcompartments of malignant gliomas beyond the areas of contrast uptake. More precise compartmental sampling may foster the development of strategies that target subtype-specific patterns of the tumor microenvironment.

Processing times were surprisingly variable among the raters. Even with semi-automatic methods, each rater has to decide when he or she has achieved satisfactory segmentation results. One may accept the two minimally required perpendicular slices as satisfactory or continue with slice-by- slice corrections. These choices may result in a considerable time and quality difference, with the consequence of resembling either computer-assisted segmentation (as intended by the automatization procedures) or of bouncing back to human control (closer to manual segmentation). We did not control for this effect since we did not provide strict guidelines about the number of interactive steps to be taken to reach the final segmentation. However, this reflects good clinical practice and may in part explain the observed time divergences and inter-rater variability. Further, we recruited the raters according to their experience in glioma reading and their oncological expertise, which might have led to different strategies in preparing the final segmentation.

Finally, segmentation performance always reflects a mixture of additional non-professional skills such as self-motivation and ability to focus on the task, as well as the influence of task-induced fatigue and a multitude of other factors, including external ones. All these potential confounders can be overcome by automated segmentation.

## Conclusions

We conclude that automated tissue compartment analysis of GBMs using fully-automated analysis tools (BT) is feasible and provides similar results to those obtained with semi-automatic ones (SB) if the clinically relevant volume and SPD measures are primarily addressed. However the two methods differ considerably in terms of Dice coefficient due to rater-dependency. In addition, BT extends the GTV by adding NETV subcompartments omitted by a single-compartment (e.g. 3D T1w contrast-enhanced sequences) analysis, as is usually performed for therapy planning. Rater independence renders the tool applicable for complex data analysis (e.g. in radiosurgery planning), for tumor growth modeling and radiomic/radiogenomic analyses. Since both methods have complementary strengths (and limitations) their usage should be related to the clinical and scientific questions under consideration.
